# Generation of a new therapeutic d-peptide that induces the differentiation of acute myeloid leukemia cells through A TLR-2 signaling pathway

**DOI:** 10.1038/s41420-024-01822-w

**Published:** 2024-01-26

**Authors:** Fei Yu, Yingshi Chen, Mo Zhou, Lingling Liu, Bingfeng Liu, Jun Liu, Ting Pan, Yuewen Luo, Xu Zhang, Hailan Ou, Wenjing Huang, Xi Lv, Zhihui Xi, Ruozhi Xiao, Wenyu Li, Lixue Cao, Xiancai Ma, Jingwen Zhang, Lijuan Lu, Hui Zhang

**Affiliations:** 1 Medical Research Institute, Guangdong Provincial People’s Hospital (Guangdong Academy of Medical Sciences), Southern Medical University, Guangzhou, Guangdong China; 2 Institute of Human Virology, Key Laboratory of Tropical Disease Control of Ministry of Education, Guangdong Engineering Research Center for Antimicrobial Agent and Immunotechnology, Zhongshan School of Medicine, Sun Yat-sen University, Guangzhou, Guangdong China; 3 Department of Hematology, The Third Affiliated Hospital, Sun-yat Sen University, Guangzhou, Guangdong China; 4 Guangzhou National Laboratory, Guangzhou, Guangdong China; 5 Department of Medical Oncology, The Third Affiliated Hospital of Sun Yat-sen University, Guangzhou, Guangdong China

**Keywords:** Drug development, Acute myeloid leukaemia

## Abstract

Acute myeloid leukemia (AML) is caused by clonal disorders of hematopoietic stem cells. Differentiation therapy is emerging as an important treatment modality for leukemia, given its less toxicity and wider applicable population, but the arsenal of differentiation-inducing agents is still very limited. In this study, we adapted a competitive peptide phage display platform to search for candidate peptides that could functionally induce human leukemia cell differentiation. A monoclonal phage (P6) and the corresponding peptide (pep-P6) were identified. Both l- and d-chirality of pep-P6 showed potent efficiency in inducing AML cell line differentiation, driving their morphologic maturation and upregulating the expression of macrophage markers and cytokines, including CD11b, CD14, IL-6, IL-1β, and TNF-α. In the THP-1 xenograft animal model, administration of d-pep-P6 was effective in inhibiting disease progression. Importantly, exposure to d-pep-P6 induced the differentiation of primary human leukemia cells isolated AML patients in a similar manner to the AML cell lines. Further mechanism study suggested that d-pep-P6 induced human leukemia cell differentiation by directly activating a TLR-2 signaling pathway. These findings identify a novel d-peptide that may promote leukemia differentiation therapy.

## Introduction

Acute myeloid leukemia (AML) is a type of fast-developing blood cancer with suppression of normal hematopoiesis and disorder of expansion hematopoietic stem cells [1]. Over the last decade, although continuing research into the pathogenesis, molecular and epigenetic landscape of AML has led to advances in the prognostication and treatment, the main “3 + 7” regimen (cytarabine plus an anthracycline) therapies of AML have not changed significantly [2]. However, as AML may be considered an elderly disease with a median age of 68 years at diagnosis [3], neither intensive chemotherapy nor allogeneic stem cell transplantation can be generally tolerated in elderly and/or unfit patients [4]. Therefore, novel, effective agents and therapeutic strategies are urgently needed for this deadly disease.

Differentiation therapy could be an effective therapy for AML. This therapeutic approach aims to remove the maturation block and allow differentiation of the immature leukemia cells. For example, the efficacies of all-trans retinoic acid (ATRA) and imatinib mesylate have been well appreciated in acute promyelocytic leukemia (APL) [5] and chronic myeloid leukemia (CML) [6], respectively, which significantly improve the clinical outcome. Undeniably, the rapid clearance of APL in response to ATRA was a hallmark success of differentiation therapy, long considered to be the milestone of this new therapeutic avenue. However, despite 40 years of extensive research, the same dramatic clinical responses have not been replicated in other diseases and drug pairing [2]. For non-APL AML patients, the relapse rates and subsequent disease-associated mortality still remain high [7]. Therefore, further investigation to develop new agents for AML differentiation therapy is urgent.

Phage display, a well-established and sophisticated in vitro evolutionary method, is a type of affinity maturation method for screening and identifying specific ligands of a variety of molecular targets [8–10]. In the present study, we employed functional phage display screening and developed a novel peptide that could induce human AML cell differentiation. A 12 amino-acid peptide QFPGFGTELPSS, named pep-P6, was identified. Both levo (l)-pep-P6 and its consistent mirror-image dextro (d)-pep-P6 were evaluated for the ability to induce the differentiation of human monocytic leukemia cells in vitro and in vivo. Mechanistically, we found that l- and d-pep-P6 could induce differentiation of human leukemia cell lines and primary human leukemia cells by triggering TLR-2 signaling. Moreover, the d-pep-P6 exhibited greater efficiency than the l-pep-P6 in inhibiting THP-1 xenograft tumor growth in vivo. These findings identify a novel d-peptide that may add to the anticancer arsenal for leukemia differentiation therapy.

## Results

### Identification of peptides-induced THP-1 differentiation by phage display

**Fig. 1:**
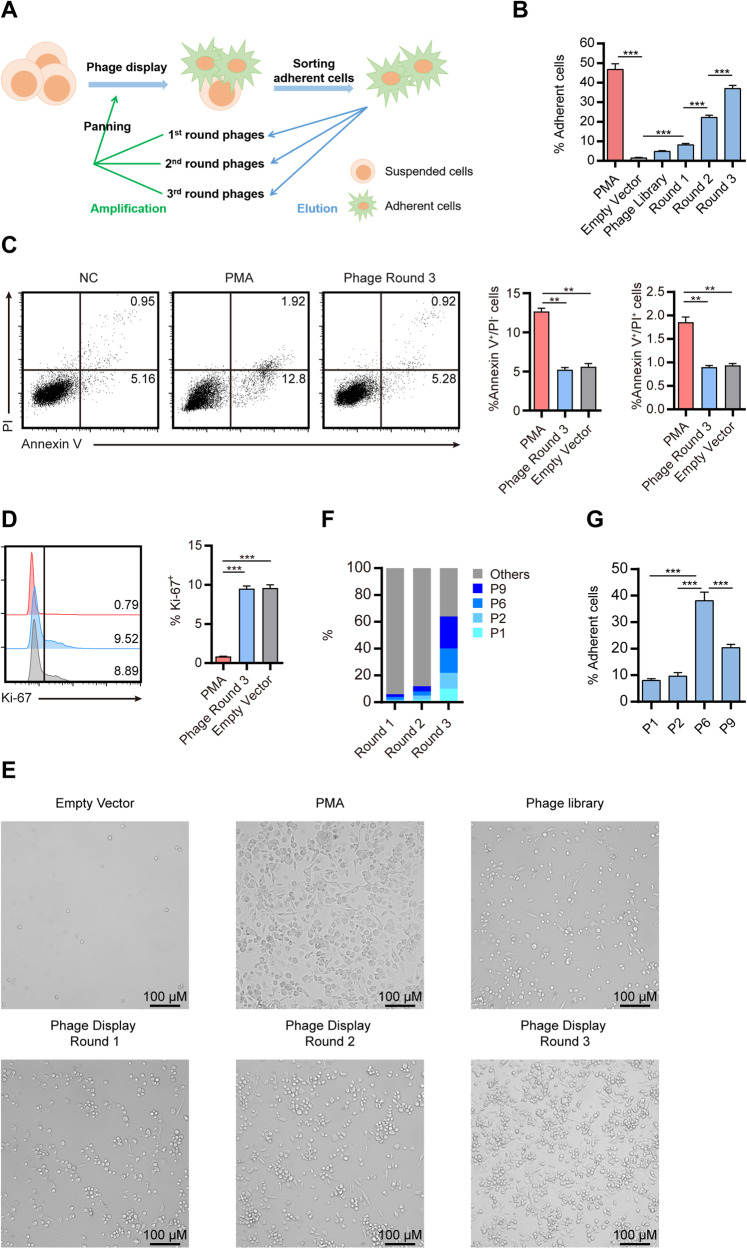
Identification of clonal phage-induced THP-1 cell differentiation by phage display. **A** Flowchart of phage display. **B** The percentage of adherent THP-1 cells induced by phage libraries or PMA. **C**, **D** THP-1 cells were treated with either PMA, phages obtained from Round 3, or an empty vector for 48 h. Flow cytometry was utilized to assess apoptosis via Annexin V/propidium iodide (PI) staining (**C**) and proliferation via Ki-67 staining (**D**). **E** THP-1 cells were stimulated with phage library or PMA for 48 h, and empty-treated cells were used as a negative control. The suspended cells were gently removed through a PBS wash. The morphological features were then visualized by phase contrast microscopy. **F** The enrichment of clonal phages during different rounds of phage display. **G** The percentage of adherent THP-1 cells induced by different clonal phages enriched in Round 3. The results are shown as the mean and SEM. ***P < 0.001 (Kruskal–Wallis test). Data are from 6 independent experiments.

To identify the candidate peptides that mediate leukemia cell differentiation, we used the THP-1 cell line as an in vitro model, which is derived from the peripheral blood of acute monocytic leukemia (M5 subtype) [11]. An in vitro functional phage screening approach was applied by incubating THP-1 cells with a Ph.D.-12 peptide phage library. After co-culture for 72 h, we separated adherent THP-1 cells, which indicated the terminal maturation of monocytic leukemia cells, from non-adherent cells. The phages associated with the adherent cells were eluted and then expanded in the bacterial culture. The expanded phages were again mixed with suspended THP-1 cells to start a second-round selection for a total of three rounds (Fig. 1A). Simultaneously, we quantified the proportion of adherent cells by flow cytometry (FACS). The proportion of adherent THP-1 cells gradually increased during the three rounds of phage display selection, suggesting the step-wise evolution and enrichment of the monoclonal phages carrying functional peptides (Fig. 1B). To ascertain whether the increased proportion of adherent cells is influenced by the proliferation or apoptosis of THP-1 cells, we conducted in vitro assays to evaluate cell death and proliferation. Taking the unstimulated group as a negative control, THP-1 cells that were stimulated with PMA or Round 3 phages were subjected to Annexin V and PI staining. Subsequently, the stained cells were analyzed using flow cytometry. The results suggested that, when compared to the unstimulated group, the proportions of apoptotic (annexin V single positive) and necrotic (annexin V and PI double positive) cells in the phage-induced group remained relatively unchanged (Fig. 1C). However, a slight increase was observed in the PMA-stimulated group (Fig. 1C). To assess cell proliferation, we also examined the expression of ki-67 in the three groups of THP-1 cells. We observed that the Round 3 phages-treated group showed no significant difference compared to the negative control, while the PMA-treated group exhibited a significant reduction in proliferation (Fig. 1D). In summary, the phages did not induce significant proliferation or cell death in THP-1 cells. The observed increase in adherent cell proportion might be attributed to the induction of cell differentiation. The morphology of THP-1 cells that were induced by phages and attached to the bottom of the plates exhibited a macrophage-like appearance, similar to the differentiation induced by phorbol 12-myristate 13-acetate (PMA) (Fig. 1E). At the end, 21 monoclonal phages were isolated, and four of them were eventually enriched during the three rounds selecting induction (Fig. 1F). Sequencing of the predominant phages (P1, P2, P6, and P9) revealed the overrepresented peptide sequences (Table 1). Each clone was expanded and tested for its ability to induce the differentiation of THP1- cells. The P6 was selected for further study as it exhibited the greatest capability for inducing THP-1 cell differentiation (Fig. 1G).

**Table 1:** Sequences of clonal phages.

Name	Sequence (5′–3′)
P1	CCQSYATLYTPL
P2	QFCGTSGEAPSP
P6	QFPGFGTELPSS
P9	QDVTLLLNFGAL

**Fig. 2:**
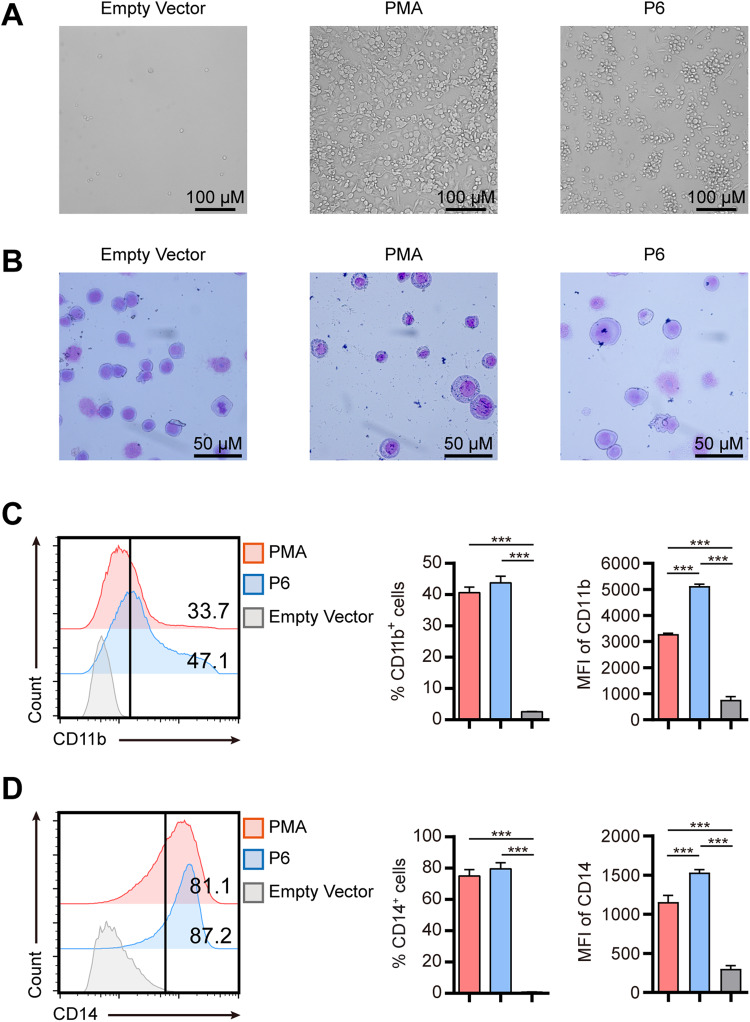
P6-induced THP-1 cells altered the morphology and enhanced macrophage surface markers. **A** THP-1 cells were stimulated with P6 or PMA for 48 h, and empty vector-treated cells were used as a negative control. The suspended cells were gently removed through a PBS wash. The morphological features were then visualized by phase contrast microscopy. **B** May-Grünwald Giemsa staining of THP-1 cells treated with either PMA, P6 or an empty vector for 48 h. **C**, **D** Cell surface markers CD11b (**C**) and CD14 (**D**) were detected by FACS after 48 h incubation with P6. The results are shown as the mean and SEM. ***P < 0.001 (Kruskal–Wallis test). MFI median fluorescent intensity. Data are from 6 independent experiments.

The differentiation-inducing function of P6 was confirmed by the phenotype and expression of cell surface markers of THP-1 cells. After exposure to P6, the adherent THP-1 cells exhibited increased cytoplasmic volume and became macrophage-like kidney shapes that were similar to the change induced by PMA (Fig. 2A). Moreover, May-Grünwald Giemsa staining showed that the adherent cells induced by P6 displayed a bluish-gray cytoplasm and a smaller, irregular nucleus compared to suspended THP-1 cells (Fig. 2B). In addition, FACS analysis showed that P6 significantly increased the expression of typical macrophage markers, such as CD11b (Fig. 2C) and CD14 (Fig. 2D). The percentage of THP-1 cells expressing surface CD11b (Fig. 2C) and CD14 (Fig. 2D) after the exposure to PMA or P6 was markedly increased compared to the empty vector treated group. Interestingly, the median fluorescence intensity (MFI) of CD11b and CD14 expression in THP-1 cells induced by P6 was even higher than that induced by PMA (1.5-fold for CD11b, ***P < 0.001 and 1.3-fold for CD14, ***P < 0.001). These results indicated that the monoclonal phage P6 could induce the differentiation of THP-1 cells.

### Generation of l-/d-peptides that induced AML cell line differentiation

**Fig. 3:**
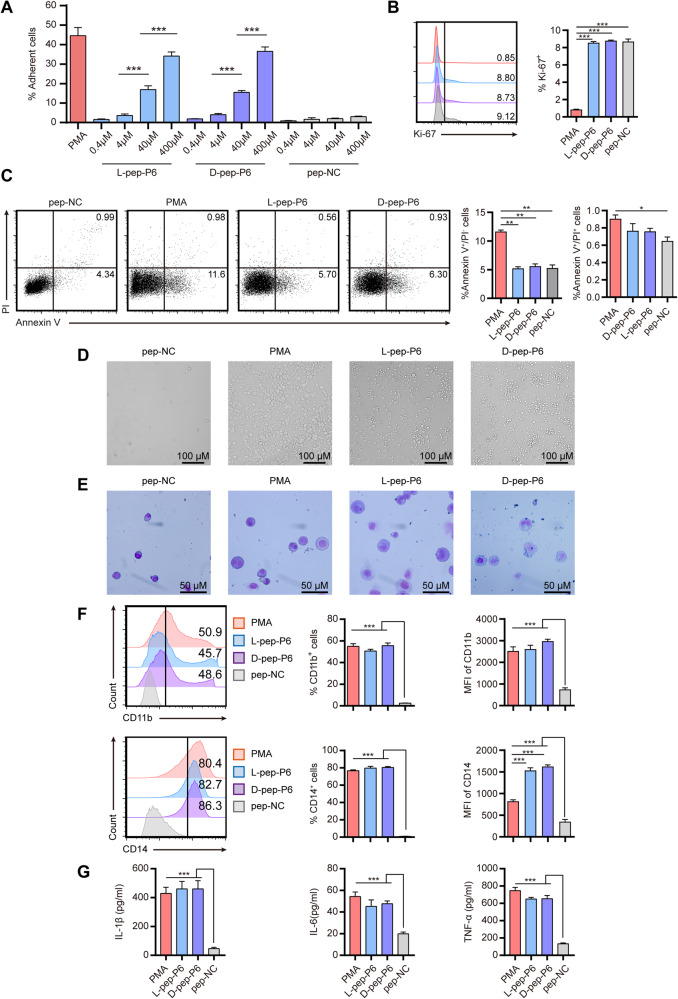
Both l- and d-pep-P6 could induce differentiation of THP-1 cells in a dose-dependent way. **A** THP-1 cells were stimulated with a range of different concentrations of l-/d-pep-P6 or pep-NC for 48 hours, with PMA-treated cells serving as a positive control. **B**–**G** THP-1 cells were stimulated with l-/d-pep-P6 (10 μg/ml), pep-NC (10 μg/ml), or PMA for 48 hours. Flow cytometry was utilized to assess proliferation via Ki-67 staining (**B**) and apoptosis via Annexin V/propidium iodide (PI) staining (**C**). **D** The suspended cells were gently removed through a PBS wash. The morphological features were then visualized by phase contrast microscopy. **E** Phenotype was detected by May-Grünwald Giemsa staining. **F** Cell surface markers CD11b and CD14 were detected by FACS. **G** Cytokine expression profiles were measured by ELISA. The supernatants were collected, and the concentrations of IL-1β, TNF-α, and IL-6 were measured. Each bar represents the mean and SEM from three independent experiments in triplicate. *P < 0.05, **P < 0.01, ***P < 0.001 (Kruskal–Wallis test). MFI median fluorescent intensity. Data are from 6 independent experiments.

In order to verify whether the peptide encoded by the monoclonal P6 could induce differentiation of THP-1, we synthesized the corresponding peptide. We first synthesized l-peptide corresponding to P6 (l-pep-P6) and verified its function by the THP-1 inducing assay. Compared with the nonspecific sequence peptide (pep-NC), we found that the l-pep-P6 was able to induce a dose-dependent cell adherence of THP-1 cell line (Fig. 3A). In addition, the l-pep-P6 also induced the macrophage-like changes in cell morphology, phenotype, and expression of cell surface markers of THP-1 cell lines (Fig. 3D–F).

The d-peptide ligand, for reasons of symmetry, binds specifically to the native l-protein with the same affinity and bears unique stereochemistry properties that lead to resistance against most of the endogenous proteolytic enzymes [12]. These characteristics make d-peptides an appealing candidate in drug development. To further develop a drug-like peptide, we evaluated the effect of the corresponding d-peptide (d-pep-P6). We found that the d-pep-P6 could also induce a dose-dependent cell adherence of the THP-1 cell line comparable to the l-pep-P6 (Fig. 3A). The increase in THP-1 cell adhesion ratio is independent of cell death or proliferation. There were no significant changes observed in cell apoptosis, necrosis, and proliferation induced by both l-pep-P6 and d-pep-P6 relative to the negative control group (Fig 3B, C). THP-1 cells induced by d-pep-P6 exhibited a macrophage-like appearance with a diffused and enlarged shape and the occurrence of a large number of intracellular vacuoles, which was also inconsistent with the effect of l-pep-P6 (Fig. 3D). In addition, May-Grünwald Giemsa staining demonstrated that both the d-pep-P6 and l-pep-P6 could promote typical monocyte/macrophage cell maturation of the THP-1 cells (Fig. 3E). Moreover, d-pep-P6 and l-pep-P6 significantly increased the expression of CD11b and CD14 on THP-1 cells, an effect that could not be seen in cells induced by pep-NC (Fig. 3F).

Furthermore, we investigated the potential of d-pep-P6 to induce differentiation across diverse AML cell lines. Employing May-Grünwald Giemsa staining and flow cytometry techniques, our findings indicated that d-pep-P6 exhibited the capacity to initiate the maturation of typical monocyte/macrophage cells within HL-60 (Supplemental Fig. 1) and U937 (Supplemental Fig. 2) cell lines. This was evident through distinct alterations in both cellular morphology (Supplemental Figs. 1A and 2A) and phenotype (Supplemental Figs. 1B and 2B), coupled with a notable increase in the proportion of CD11B and CD14 positive cells (Supplemental Figs. 1E and 2E). Notably, these outcomes are in concordance with the observations made in THP-1 cells.

In addition to the changes in morphology and phenotype, we further verified the differentiation of peptide-stimulated THP-1 cells by their cytokine secretion ability. The concentrations of IL-1β, IL-6, and TNF-α in the supernatant of cell culture were measured by ELISA. THP-1 cells treated with the d-pep-P6 and l-pep-P6 produced profoundly more amounts of IL-1β, TNF-α, and IL-6 compared to those treated with pep-NC (Fig. 3G). Together, the results of these phenotypic and functional assays suggest that l-pep-P6 and d-pep-P6 can effectively induce THP-1 cell differentiation into macrophage-like cells.

### l-pep-P6 and d-pep-P6 peptides suppressed tumor growth in a THP-1 xenograft model

**Fig. 4:**
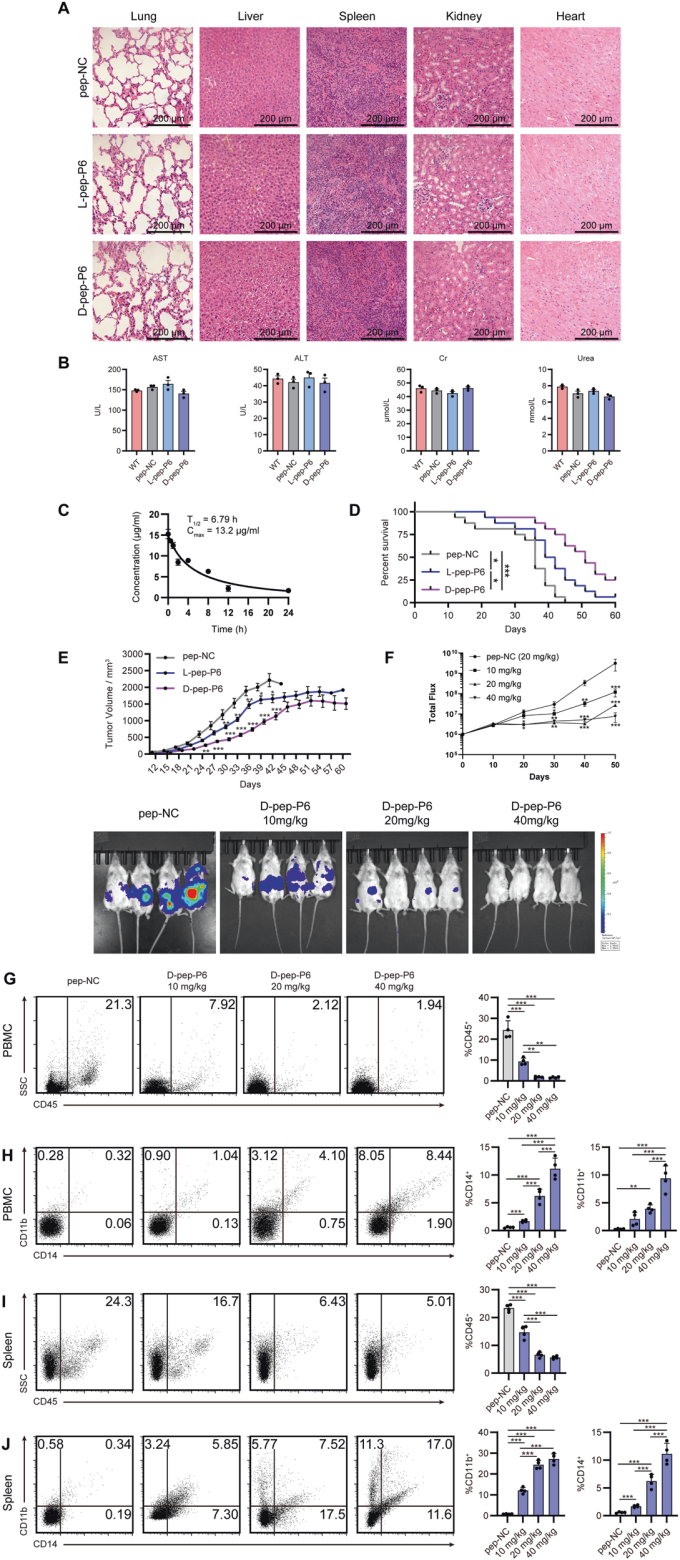
l-pep-P6 and d-pep-P6 inhibited disease progression and prolonged the animals’ survival in THP-1 xenograft mice models. **A** Multiple organs of NOD/SCID mice from L-/d-pep-P6 and pep-NC treated groups (at a dose of 200 mg/kg) were fixed in 4% formaldehyde for hematoxylin and eosin staining (*n* = 3 mice per group). **B** Glucose and serum biochemical indices, such as Aspartate Aminotransferase (AST), Alanine Aminotransferase (ALT), Creatinine (Cr), and urea were detected (mean ± SEM, *n* = 3 mice per group). **C** The plasma concentration-time curve of d-pep-P6 after intravenous administration of 20 mg/kg in NOD/SCID mice (mean ± SEM, *n* = 3 mice per time point). **D** Survival comparison of the 20 mg/kg l-/d-pep-P6 and 20 mg/kg pep-NC treated groups in NOD/SCID THP-1 tumor-bearing mice (*n* = 16 mice per group). **E** Average tumor volumes of NOD/SCID mice treated with 20 mg/kg pep-NC or 20 mg/kg l-/d-pep-P6 (*n* = 16 mice per group). **F** NSG mice were intravenously injected with THP-1 cells containing a luciferase reporter gene via the tail vein. The mice were treated with different concentrations of d-pep-P6, with a control group receiving 20 mg/kg pep-NC. The total burden of disease was calculated using bioluminescence with the in vivo imaging system (IVIS) measuring total flux from a region of interest (ROI) drawn over the entire animal. **G**–**J** At day 50, mice PBMC (**G** and **H**) and splenic (**I** and **J**) samples were collected, and the expressions of surface markers on THP-1 cells were analyzed by FACS. **G**, **J** CD45 were detected by FACS. **H**, **J** CD11b and CD14, after gating on CD45^+^ cells, were detected by FACS. *P < 0.05; **P < 0.01; ***P < 0.001 (Kruskal–Wallis test). Data are from 3 independent experiments.

Based on the above in vitro findings, we next tested whether the peptides could suppress the growth of leukemia cells in vivo. To this end, NOD/SCID mice were subcutaneously inoculated with THP-1 cells. l-pep-P6, d-pep-P6, or pep-NC was injected intravenously into NOD/SCID mice (*n* = 3 per group) at a 10-fold treatment dose (200 mg/kg) every 3 days. We first examined the toxicity of these peptides in vivo. After 30 days of indicated treatments, the major organs, including the heart, kidney, spleen, liver, and lung, were isolated and stained with hematoxylin and eosin. No histological changes in major organs were detectable in the peptide-treated groups when compared with the untreated group (Fig. 4A). We further examined the glucose and serum biochemical indices, such as aspartate aminotransferase (AST), alanine aminotransferase (ALT), creatinine (Cr), and urea. All of the indices showed no significant differences between the peptide-treated and control groups, suggesting that these peptides may not lead to significant abnormality in liver and kidney functions (Fig. 4B). Mice from all groups did not exhibit any signs of toxicity, such as areas of redness or swelling, impaired movement, and posture, indigestion or diarrhea, and agitation. These preliminary results suggested the low toxicity of both l-pep-P6 and d-pep-P6 peptides.

In addition, we administered a therapeutic dose of d-pep-P6 (20 mg/kg) via intravenous tail injection into NOD/SCID mice (*n* = 3 per group). Serum samples were collected at various time points, and the content of d-pep-P6 was quantitatively analyzed using high-performance liquid chromatography coupled with mass spectrometry (Supplemental Fig. 3). We observed that d-pep-P6 exhibited remarkable resistance to proteolysis and remained highly stable, with an intravenous elimination half-life (*T*_1/2_) of 6.79 h and a maximum concentration (*C*_max_) of 13.2 μg/mL (Fig. 4C).

Subsequently, the in vivo therapeutic efficacies of l-pep-P6 and d-pep-P6 were evaluated. We acquired NOD/SCID mice (*n* = 16 per group), administered subcutaneous injections of THP-1 cells, and observed the pace of tumor growth. The median survival time was 36 days, 41 days, and 51 days for the pep-NC control group, the l-pep-P6-treated group, and the d-pep-P6-treated group, respectively (Fig. 4D). The d-pep-P6 administration and, to a lesser extent, the l-pep-P6 treatment significantly prolonged the survival of animals when compared to pep-NC-treated controls. In the l-pep-P6 treated group, the suppression of tumor growth was statistically significant from day 30 after the first drug exposure (*P < 0.05). Meanwhile, d-pep-P6 remarkably suppressed tumor growth from day 24 after the first administration (**P < 0.01; Fig. 4E). Moreover, there were no statistically significant differences in body weight between the control and peptide-treated mice (Supplemental Fig. 4). These data suggested that d-pep-P6 administration could suppress THP-1 xenograft tumor growth in vivo.

To further investigate the therapeutic window of d-pep-P6 and its potential for inducing cellular differentiation in vivo, we re-established a leukemia model by injecting THP-1 cells via the tail vein. Immunodeficient female NOd-SCID/IL2γ−/− (NSG) mice (4–6 weeks old, *n* = 4 per group) were exposed to a radiation dose of 250 cGy to enhance engraftment. Subsequently, 2 × 10^6^ THP-1 AML cells containing the luciferase reporter gene were introduced into the mice via the tail vein. In the treatment group, d-pep-P6 was administered at doses of 10, 20, and 40 mg/kg through the tail vein every 3 days, while a control group received pep-NC injections at the same time points. These mice were subsequently monitored using bioluminescence imaging to assess the progression of the disease. After administering d-pep-P6 at dosages of 10, 20, and 40 mg/kg, a noteworthy dose-dependent reduction in the progression of AML was observed, particularly evident in the 40 mg/kg treatment group, which exhibited minimal disease progression. This reduction was supported by a significant decrease in tumor burden quantified using in vivo imaging system (IVIS) imaging (Fig. 4F). These findings demonstrate the efficacy of d-pep-P6 in reducing the AML burden and its potential within a relatively broad range of drug concentrations, making it suitable for clinical drug development.

To assess the in vivo induction potential of d-pep-P6, both peripheral blood mononuclear cells (PBMC) and spleen were collected from each mouse in the four groups on Day 50, and subsequently subjected to analysis using flow cytometry. A significant decrease in the proportion of human CD45-positive cells was observed in the groups treated with the peptide, particularly noticeable in the 40 mg-treated groups (Fig. 4G, I). This observation is consistent with the findings from IVIS imaging.

We conducted further analysis of the surface markers on these CD45^+^ human AML cells and observed significant increases in the proportion of CD14 and CD11 B-positive cells. The increases were more pronounced in the high-dose groups (Fig. 4H, J). These findings suggest that d-pep-P6 effectively mitigates AML progression in a dose-dependent manner.

### d-pep-P6 induces differentiation of THP-1 cells through TLR-2 signaling pathway

**Fig. 5:**
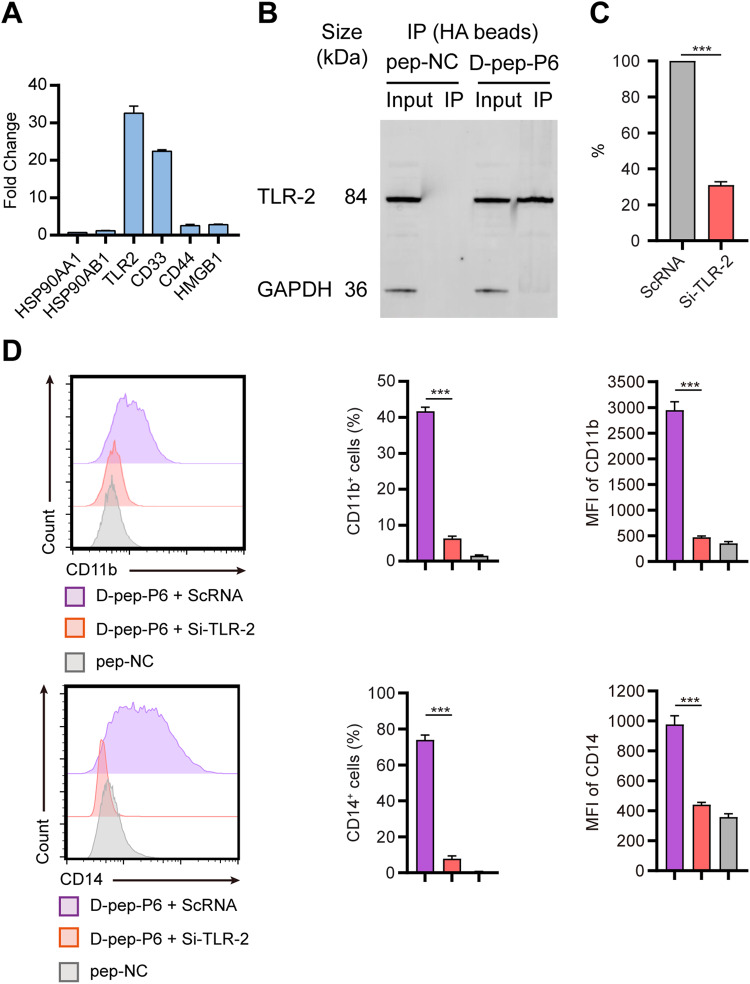
d-pep-P6 induced differentiation of THP-1 cells through TLR-2 signaling pathway. **A** Expression of the candidate proteins was measured by real-time PCR after induction by d-pep-P6, compared to the untreated group. **B** Western blot analysis of co-IP d-pep-P6 with TLR-2 using anti-TLR-2 mAb. **C** Knock out the efficiency of TLR-2 by siRNA was measured by real-time PCR. A scrambled SiRNA (ScRNA) was used as a control. **D** d-pep-P6 were incubated with THP-1 cells that knock out TLR-2 by siRNA or ScRNA, and pep-NC treated cells were used as a negative control. Cell surface markers CD11b and CD14 were detected by FACS after 48 h incubation. Each bar represents the mean and SEM from three independent experiments in triplicate. ***P < 0.001 (Kruskal–Wallis test). MFI median fluorescent intensity. Data are from 3 independent experiments (**A**–**C**) or 6 independent experiments (**D**).

To identify the receptor on the cellular membrane interacting with d-pep-P6, we synthesized d-pep-P6 and pep-NC with an HA label on the C-terminal. THP-1 cells were treated with HA-label d-pep-P6 or HA-label pep-NC overnight. The total lysate was separated and purified with anti-HA beads by western blotting. The distinct gel bands, which contained indicated peptide-binding proteins, as well as control gel bands, proceeded to in-gel digestion, followed by nanoscale LC-MS/MS to characterize the digested peptides. Thirty-nine candidate membrane proteins that might bind to pep-P6 were identified (Table 2). Among these, six of the candidate proteins were closely related to leukemia therapy and differentiation, and thus, their expression levels in THP-1 cells were further verified by real-time PCR assays. The results showed that the upregulation of toll-like receptor-2 (TLR-2) and CD33, among the six potential d-pep-P6 receptors, was most remarkable in THP-1 cells treated with pep-P6 compared to cells treated with control peptides (Fig. 5A). Co-immunoprecipitation (Co-IP) results revealed that only TLR-2 has a direct interaction of d-pep-P6 (Fig. 5B). Next, knockdown experiment was then performed using siRNA against TLR-2 or a scrambled siRNA (ScRNA) as control. The knockdown efficiency was about 70% in THP-1 cells (Fig. 5C). TLR-2 silencing decreased the d-pep-P6–induced surface expression of CD14 and CD11b on THP-1 cells by a 9.4-fold and a 6.6-fold, respectively (Fig. 5D). These results indicated that d-pep-P6 directly triggered TLR-2 signaling and induced differentiation of THP-1 cells.

**Table 2:** Proteins uniquely enriched in the pep-P6-binding group.

Accession	Score	Mass	Description
A0A024R5U5	275	86140	ADAM metallopeptidase domain 10, isoform CRA_b OS=Homo sapiens GN = ADAM10 PE = 4 SV = 1
A0A024R8S5	3203	57480	Protein disulfide-isomerase OS=Homo sapiens GN = P4HB PE = 2 SV = 1
A0A024RD80	6052	83554	Heat shock protein 90 kDa alpha (Cytosolic), class B member 1, isoform CRA_a OS=Homo sapiens GN = HSP90AB1 PE = 3 SV = 1
A0A0S2Z4S4	140	90920	Toll-like receptor 2 isoform 1 (Fragment) OS=Homo sapiens GN = TLR2 PE = 2 SV = 1
A8K401	863	29843	Prohibitin, isoform CRA_a OS=Homo sapiens GN = PHB PE = 2 SV = 1
O15031	154	207734	Plexin-B2 OS=Homo sapiens GN = PLXNB2 PE = 1 SV = 3
O75787	311	38983	Renin receptor OS=Homo sapiens GN = ATP6AP2 PE = 1 SV = 2
P05107	870	87976	Integrin beta-2 OS=Homo sapiens GN = ITGB2 PE = 1 SV = 2
P06733	2780	47481	Alpha-enolase OS=Homo sapiens GN = ENO1 PE = 1 SV = 2
P07204	276	63083	Thrombomodulin OS=Homo sapiens GN = THBD PE = 1 SV = 2
P07355	1158	38808	Annexin A2 OS=Homo sapiens GN = ANXA2 PE = 1 SV = 2
P07900	5679	85006	Heat shock protein HSP 90-alpha OS=Homo sapiens GN = HSP90AA1 PE = 1 SV = 5
P08311	199	29161	Cathepsin G OS=Homo sapiens GN = CTSG PE = 1 SV = 2
P08758	2046	35971	Annexin A5 OS=Homo sapiens GN = ANXA5 PE = 1 SV = 2
P09382	331	15048	Galectin-1 OS=Homo sapiens GN = LGALS1 PE = 1 SV = 2
P09429	746	25049	High mobility group protein B1 OS=Homo sapiens GN = HMGB1 PE = 1 SV = 3
P09525	3172	36088	Annexin A4 OS=Homo sapiens GN = ANXA4 PE = 1 SV = 4
P11021	3667	72402	78 kDa glucose-regulated protein OS=Homo sapiens GN = HSPA5 PE = 1 SV = 2
P13612	237	116252	Integrin alpha-4 OS=Homo sapiens GN = ITGA4 PE = 1 SV = 3
P14384	145	50938	Carboxypeptidase M OS=Homo sapiens GN = CPM PE = 1 SV = 2
P16070	210	82001	CD44 antigen OS=Homo sapiens GN = CD44 PE = 1 SV = 3
P17655	1093	80800	Calpain-2 catalytic subunit OS=Homo sapiens GN = CAPN2 PE = 1 SV = 6
P17931	213	26193	Galectin-3 OS=Homo sapiens GN = LGALS3 PE = 1 SV = 5
P20138	188	40313	Myeloid cell surface antigen CD33 OS=Homo sapiens GN = CD33 PE = 1 SV = 2
P30040	399	29032	Endoplasmic reticulum resident protein 29 OS=Homo sapiens GN = ERP29 PE = 1 SV = 4
P30101	1940	57146	Protein disulfide-isomerase A3 OS=Homo sapiens GN = PDIA3 PE = 1 SV = 4
P34741	129	22203	Syndecan-2 OS=Homo sapiens GN = SDC2 PE = 1 SV = 2
P54578	509	56489	Ubiquitin carboxyl-terminal hydrolase 14 OS=Homo sapiens GN = USP14 PE = 1 SV = 3
P54652	1019	70263	Heat shock-related 70 kDa protein 2 OS=Homo sapiens GN = HSPA2 PE = 1 SV = 1
Q00839	504	91269	Heterogeneous nuclear ribonucleoprotein U OS=Homo sapiens GN = HNRNPU PE = 1 SV = 6
Q12846	374	34273	Syntaxin-4 OS=Homo sapiens GN = STX4 PE = 1 SV = 2
Q12904	712	34616	Aminoacyl tRNA synthase complex-interacting multifunctional protein 1 OS=Homo sapiens GN = AIMP1 PE = 1 SV = 2
Q12907	793	40545	Vesicular integral-membrane protein VIP36 OS=Homo sapiens GN = LMAN2 PE = 1 SV = 1
Q13641	161	46573	Trophoblast glycoprotein OS=Homo sapiens GN = TPBG PE = 1 SV = 1
Q92692	187	58162	Nectin-2 OS=Homo sapiens GN = NECTIN2 PE = 1 SV = 1
Q9BS26	186	47341	Endoplasmic reticulum resident protein 44 OS=Homo sapiens GN = ERP44 PE = 1 SV = 1
Q9Y490	288	271766	Talin-1 OS=Homo sapiens GN = TLN1 PE = 1 SV = 3
V9HW88	647	48283	Calreticulin, isoform CRA_b OS=Homo sapiens GN = HEL-S-99n PE = 2 SV = 1
V9HW90	260	56791	Epididymis luminal protein 75 OS=Homo sapiens GN = HEL-75 PE = 2 SV = 1

### d-pep-P6 induces the differentiation of leukemia cells derived from AML patients

**Fig. 6:**
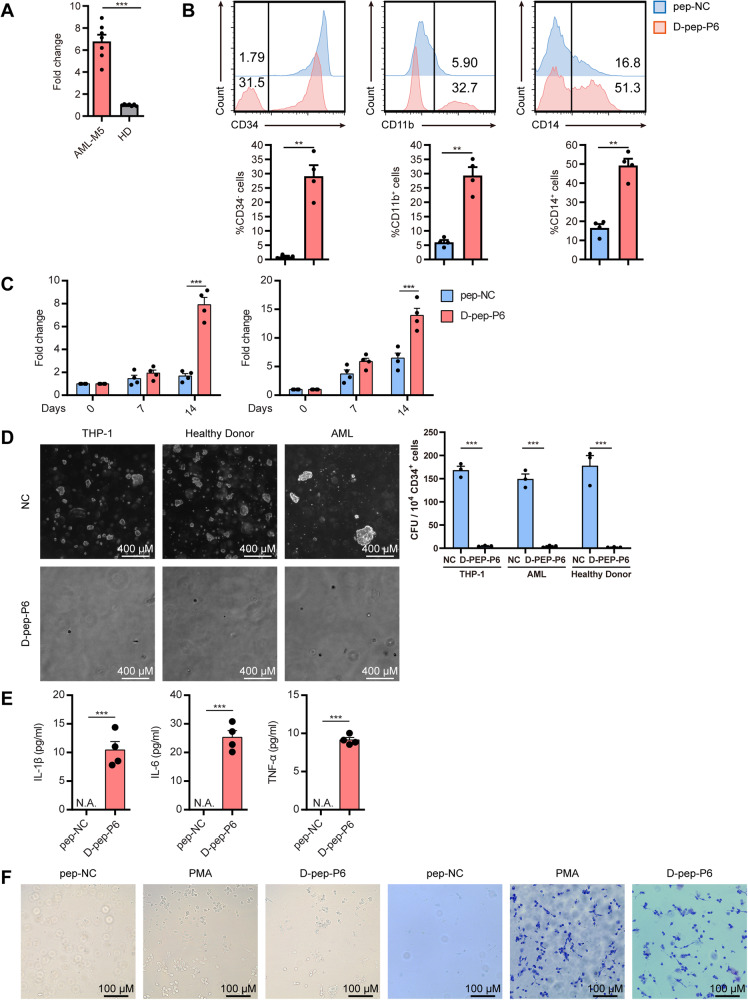
d-pep-P6 could induce differentiation of leukemia cells that derived from M5 AML individuals. **A** Expression of TLR-2 of peripheral blood CD34^+^ cells isolated from M5 AML (M5) individuals and healthy donors (HD) was measured by real-time PCR. ***P < 0.001 (*t*-test). *n* = 7 individuals per group. **B** Cell surface markers CD34, CD11b, and CD14 were detected by FACS after 14 days of incubation with d-pep-P6 or pep-NC (10 μg/ml). **P < 0.05 (paired *t*-test). *n* = 4 individuals per group. **C** Expression of CD11b and CD14 of leukemia cells were exposed to 10 μg/ml d-pep-P6 or 10 μg/ml pep-NC were measured by real-time PCR at Day 0, Day 7, and Day14 ***P < 0.001 (paired *t*-test). *n* = 4 individuals per group. **D** The colony-forming efficiency of CD34 cells from AML donors, healthy donors, and THP-1 cells was assessed. These cells were incubated with d-pep-P6 or pep-NC (10 μg/ml) for 15 days and subsequently examined using light microscopy. ***P < 0.001 (paired *t*-test). *n* = 3 individuals per group. **E** Cy*t*okine expression profiles were measured by ELISA. Leukemia cells were exposed to d-pep-P6 or pep-NC for 14 days. The supernatants were collected, and the concentrations of IL-1β, TNF-α, and IL-6 were measured. Results were shown as mean and SEM. ***P < 0.001 (paired *t*-test). *n* = 4 individuals per group. **F** CD34^+^ cells isolated from individuals with M5 AML (M5) were incubated with d-pep-P6 (10 μg/ml), pep-NC (10 μg/ml), or PMA for 48 h. After incubation, suspended cells were gently removed through a PBS wash, and the morphological features were visualized using phase-contrast microscopy. The phenotype was detected through May-Grünwald Giemsa staining. *n* = 3 individuals per group.

To investigate whether the peptide could induce the differentiation of primary leukemia cells, we recruited 7 M5 AML patients. The CD34^+^ cells were isolated from the peripheral blood of the M5 AML individuals by FACS. RT-PCR was performed to detect TLR-2 expression of leukemia cells. The results revealed that CD34^+^ cells derived from M5 AML patients upregulated TLR-2 expression sevenfold relative to CD34^+^ cells from healthy donors (normalized to expression of β-actin, Fig. 6A).

The freshly isolated CD34^+^ AML cells were divided into two groups for long-term and short-term culture, respectively, and then treated with d-pep-P6 to test whether the peptide could induce monocyte/macrophage differentiation of primary AML cells. For the long-term culture, the results indicated that exposure to d-pep-P6 significantly reduced the expression of the stem-cell marker CD34 on primary AML cells (Fig. 6B). Furthermore, d-pep-P6 exhibited a significant inhibitory effect on the colony-forming activity of THP-1 cells and CD34 cells derived from both AML and healthy donors (Fig. 6D). This suggests a decline in the blast phenotype characteristics of cancer cells. In accordance, the d-pep-P6 treatment increased the expression of monocyte/macrophage maturation markers CD14 and CD11b (Fig. 6B, C). In addition, the l-pep-P6 also induced the macrophage-like changes in cell morphology, phenotype. ELISA confirmed that the d-pep-P6 drove primary AML cells to increase the secretion levels of IL-6, IL-1β, and TNF-α (Fig. 6E). Furthermore, in the case of short-term culture, l-pep-P6 also induced macrophage-like changes in both cell morphology and phenotype (Fig. 6F). Altogether, these data indicated that d-pep-P6 is able to promote the monocyte/macrophage differentiation of primary AML cells.

## Discussion

Acute myeloid leukemia (AML) is characterized by arrested differentiation of hematopoietic stem cells. Differentiation therapy could be an effective treatment for AML, but the therapeutic agents are still limited. Identifying novel differentiation-inducing drugs is necessary for striding forward in differentiation therapy for AML. In the current study, we employed functional phage display screening and developed a novel peptide that could induce differentiation in human leukemia cells. A novel d-peptide, named d-pep-P6, was identified. This d-peptide might function as a TLR-2 agonist and exhibit marked capability to drive human AML cell differentiation both in culture and in a xenograft mouse model. Therefore, these results identify a novel d-peptide that may promote leukemia differentiation therapy.

Differentiation therapy using ATRA for APL has gained great success in the clinic. However, this success of differentiation therapy has not yet been replicated for non-APL subtypes of AML patients. Chemotherapy with allogeneic stem cell transplantation is still the most effective treatment for non-APL AML. More recently, several novel differentiation-inducing agents have gained regulatory approval, such as inhibitors of B-cell lymphoma 2 (BCL2) [13], inhibitors of isocitrate dehydrogenase (IDH1 or IDH2) [14, 15], and inhibitors of lysine-specific demethylase-1 (LSD1) [16] and vitamin D derivative [17]. However, the therapeutic utility has been limited in the clinic, which presses the need for developing novel agents [18–20]. Selection from phage display libraries provides a fast and convenient way to develop novel peptides with high specificity and affinity of protein targets, making it an important way to discover novel drugs. In the present study, we screened peptides that could induce differentiation of leukemia cells by using a functional phage display technology. The findings demonstrate that phage displays not only can identify the interaction of peptides and their potential receptors but can screen for peptides that trigger certain cellular behaviors or molecular signaling in target cells.

Drawing from previous research findings, it has been reported that certain AML treatment protocols, which combine RA (all-trans retinoic acid) with conventional anthracycline-based frontline chemotherapy, have yielded notable improvements in survival rates, particularly in cases where a mutation in the gene responsible for encoding nucleophosmin 1 (NPM1) is present [21, 22]. Therefore, a combinational approach warrants consideration. Additional chemotherapy agents or targeted therapeutics can be administered in tandem with d-pep-P6 to further enhance the prognosis of patients.

Compared to small molecules or antibodies, peptides usually have high protein-binding specificity and less chance of unintended interaction with the immune system [23]. However, the susceptibility of l-peptides to proteolysis limits their applications. Considering that d-peptides are more resistant to proteolysis and have longer circulation half-time in vivo [12, 24, 25], we evaluated the efficacy of both the l- and d-pep-P6. We found that although the two peptides showed comparable ability to induce THP-1 cell differentiation in vitro, d-pep-P6 was more effective in suppressing the tumor growth in vivo and increasing the animals’ survival. As chemical synthesis is the only method to generate d-peptides, mirror image phage display is limited to the protein targets that are chemically accessible and, thus, has rarely been practiced in drug discovery [26]. Our findings provide a convenient way to bridge the advantages of phage display and d-peptide drug delivery systems [12]. Furthermore, when compared to other compounds or TLR-2 agonists, d-pep-P6, given its short peptide nature, lends itself readily to expression by CAR-T cells, offering a promising avenue for targeted therapy.

Mechanistic studies indicate that these peptides function by interacting with TLR-2. In recent studies, TLRs were considered as potential therapeutic targets in hematologic malignancies [27–29]. Of note, stimulation of cell-surface TLR-2 and TLR-4 on normal hematopoietic cells leads to the differentiation and proliferation of hematopoietic stem cells and myeloid progenitor cells [30]. It has also been shown that a wide range of TLRs is expressed in AML cells, which may be associated with the pathogenesis and development of AML [31]. Considering TLR2 expression on hematopoietic stem cells and myeloid progenitor cells, d-pep-P6 may exert an influence on normal cells. This was substantiated by CFU assays, where d-pep-P6 significantly inhibited the colony-forming activity of CD34^+^ cells derived not only from AML but also from healthy donors (Fig. 6D). Despite the potential risk of inducing differentiation in normal hematopoietic stem cells, d-pep-P6, even when administered at a 10-fold treatment dose, did not cause damage to the major organs of the mice (Fig. 4A, B). This observation is promising in alleviating concerns regarding potential off-target effects. Additionally, we also found that CD34^+^ cells derived from M5 AML patients upregulated TLR-2 expression by sevenfold in relative to CD34^+^ cells from healthy donors. Co-IP results revealed a direct interaction of d-pep-P6 with TLR-2. Knockdown of TLR-2 by siRNA hampered THP-1 cell maturation induced by both L- and d-pep-P6. These results suggest that the l- and d-pep-P6 peptides induce AML leukemia differentiation in a TLR-2-dependent way. Therefore, our data suggests that d-pep-P6 functions as a TLR-2 agonist in the leukemic cells.

In summary, the present study identifies that d-pep-P6 can selectively induce the differentiation of both human leukemia cell lines and primary human leukemia cells by activating TLR-2 signaling. In a THP-1 xenograft model, d-pep-P6 was effective in inhibiting tumor growth in vivo. Further efforts to understand the mechanisms by which d-pep-P6 induces differentiation of human AML leukemia cells in a more humanized, immune component model may provide valuable information that helps identify molecular subgroups of AMLs and potentially other hematologic malignancies that might be responsive to d-pep-P6 treatment. Additionally, exploring the potential synergy of d-pep-P6 with other chemotherapy drugs or CAR-T cell therapy may hold significant potential for improving the overall prognosis and offer promising prospects for AML patients.

## Materials and methods

### Human subjects

Peripheral blood was obtained from seven patients with M5 AML and from seven healthy donors. The blood CD34^+^ cells were isolated by FACS (Table 3). All of these individuals were recruited from The Third Affiliated Hospital of Sun Yat-Sen University. All human samples were anonymously coded in accordance with the local ethical guidelines (as stipulated by the Declaration of Helsinki). The written informed consent was provided by all study participants, and the protocol was approved by the institutional review board (IRB) of The Third Affiliated Hospital of Sun Yat-Sen University (Guangzhou, China).

**Table 3:** Fluorochrome-conjugated antibodies used for flow cytometry.

Antigen	Specificity		Fluorochrome	Clone	Supplier
CD34	Human		FITC	561	BioLegend
CD45	Human		APC/Cyanine7	HI30	BioLegend
CD14	Human		PE	63D3	BioLegend
CD11b	Human		APC	ICRF44	BioLegend
Ki-67	Human		PE	Ki-67	BioLegend

*PE* phycoerythrin, *FITC* fluorescein isothiocyanate, *APC* allophycocyanin. The antibodies were diluted in PBS containing 1% FCS. For flow cytometric staining, 5 µl of CD34, CD45, CD14, CD11b, or Ki-67 was utilized for each test per million cells in a staining volume of 100 µl.

### Cell line and cell culture

The THP-1, U937, and HL-60 cell lines were purchased from ATCC. THP-1, HL-60, and U937 cells were cultured in RPMI 1640 containing 2 mM glutamine, 100 IU/ml penicillin and 100 μg/ml streptomycin (Sigma), supplemented with 10% fetal bovine serum (FBS; Gibco). Cell cultures were maintained at 37 °C, 5% CO_2_ at 0.8–1 × 10^5^ cells/ml. For the initiation of differentiation in THP-1, HL-60, U937, and primary AML cells, the cells were seeded into 12-well tissue culture plates at 1 × 10^6^ cells/well. Subsequently, these cells were exposed to the indicated clonal phages (10^10^ virions per well), peptides (10 μg/ml), or 100 nM PMA (Sigma-Aldrich, MO, USA).

### Cell proliferation and apoptosis assay

To assess proliferation, Ki-67 was detected using FACS after 48 h of cell culture exposed to a specific stimulation. Following the manual instruction, the target cells were incubated with 70% cold ethanol at −20 °C for 1 h, washed, and then suspended at a concentration of 1.0 × 10^7^/ml in BioLegend Cell Staining Buffer. Subsequently, 100 µl cell suspension was incubated with the 5 µl Ki-67 antibody for flow cytometric analysis.

APC Annexin V Apoptosis Detection Kit with PI (BioLegend, Catalog # 640932, CA, USA) was employed to evaluate cell apoptosis. Target cells were washed twice with cold BioLegend’s Cell Staining Buffer, then suspended in Annexin V Binding Buffer at a concentration of 0.25–1.0 × 10^7^ cells/ml. To 100 µL of the cell suspension, 5 µL of APC Annexin V and 10 µL of Propidium Iodide Solution (PI) were added. The suspension was gently vortexed and incubated in the dark for 15 minutes at room temperature. Finally, 400 µL of Annexin V Binding Buffer was added to each tube, and the samples were analyzed by flow cytometry. Annexin V binds exclusively to cells expressing phosphatidylserine on the outer cell membrane layer, while PI selectively stains the DNA of cells with compromised membranes. This distinction allows for the discrimination between live cells (unstained by either fluorochrome), apoptotic cells (stained solely with annexin V), and necrotic cells (stained with both annexin and PI).

### Cell differentiation induction

Cells were cultured according to the method described above. As the adherent cell numbers reached a peak at 48 h and declined after 72 h, we chose 48 h as the incubation time in the following experiments [32]. Following the incubation period, cells within each were washed 3 times with PBS. Cells were considered to be adherent if they resisted the vigorous shake of the culture flask and washing by pipetting with a normal growth medium [33]. To assess the ratio of adherent cells, an equal amount of FITC beads (BioLegend, Catalog # 424601, CA, USA) was separately introduced to both suspended and adherent cells. Subsequently, the proportions of FITC beads were measured using FACS. The ratio of adherent cells is then computed by comparing FITC-bead ratios present within the two cell populations.

Differentiation status was also assessed by morphological observation of the cells and by FACS detection of cell surface CD11b and CD14 expression (Table 3). FcR Blocking Reagent (Miltenyi Biotec) was employed to prevent undesired antibody binding and enhance the staining specificity of cell surface antigens.

### Phage display

Phage display procedures were carried out as described in detail in the manual provided by the Ph.D.-12 Phage Display Peptide Library kit (E8110S, New England Biolabs) with modifications. In brief, phages from the Ph.D.-12 Phage Display Peptide Library was added to the culture of THP-1 cells. After a 72 h-culture, the adherent THP-1 cells were separated from the non-adherent cells. The phages associated with the adherent cells were eluted and then expanded in the bacterial culture. The expanded phages were purified and again mixed with suspended THP-1 cells to start a second-round selection for a total of three rounds. After the third round of biopanning, each of the individual phages from the eluted pool was amplified. The phage pellet was concentrated by 20% PEG/2.5 M NaCl. The single-stranded DNA of phage clones was then extracted by NaI Buffer (10 mM Tris-HCl pH 8.0, 1 mM EDTA, 4 M NaI) and ethanol as described in the instruction manual of Ph.D. Phage Display Libraries. A specific primer corresponding to the pIII gene sequence (5′-CCC TCA TAG TTA GCG TAA CG-3′) was used for sequencing to identify the clone of phages.

### May-Grünwald Giemsa staining

The THP-1 cells with indicated treatments were cytospanned and stained with May-Grünwald for 5 min and Giemsa for 30 min. Then, the cell smears were washed with water and air-dried, followed by observation under Olympus BX51 optical microscopy (Olympus, Tokyo, Japan).

### H&E staining

The mice were categorized into three groups, including the pep-NC group, the d-pep-P6 group, and the l-pep-P63 group (*n* = 3 per group). These groups received a dosage that was ten times the standard therapeutic amount via the tail vein. Major organs of mice from different treated groups were fixed in 4% formaldehyde for hematoxylin and eosin staining. Following a hydration process, the tissues were stained with Harris hematoxylin for 15 min. After a 3-min wash, the slides were briefly immersed in 1% hydrochloric acid in 75% ethanol for 30 s. Prior to dehydration, the tissues were stained with eosin for 10 min. Lastly, the slides were placed in xylene and mounted using a mounting medium.

### In vivo pharmacokinetic assay

Female NOD/SCID mice weighing between 18 and 20 g were utilized for the pharmacokinetic analysis of d-pep-p6. In the plasma, d-pep-p6 pharmacokinetic profile analysis, groups of mice (*n* = 3) were administered a single intravenous dose of 20 mg/kg. Blood samples were collected at specified time intervals and subsequently centrifuged at 8000 rpm for 10 min. The resulting plasma was mixed with CH_4_O in a 3:1 (v/v) ratio and centrifuged once more at 13,000 rpm for 10 min. The supernatant was then analyzed using RP-HPLC to determine parameters such as the maximum plasma concentration (*C*_max_) and elimination half-life (*T*_1/2_).

### Xenograft animal model

NOD/SCID mice at 4 weeks old were purchased from Vital River Laboratories (VRL, Beijing, China). All animal studies were conducted according to protocols approved by the University’s Institutional Animal Care and Use Committee (IACUC). The mice were subcutaneously inoculated with 2 × 10^6^ THP-1 cells and then randomly divided into three groups (*n* = 16 per group). Five days after tumor inoculation, the mice were intravenously injected with either d-/l-pep-P6 at 20 mg/kg every three days or an equal volume of vehicle. The mice were monitored for tumor growth, body weight, and time of death every 3 days. Tumors were measured in two dimensions (length and width) using callipers, and the tumor volume was calculated using the formula: tumor size (mm^3^) = 0.5 × (length × width × width). Mice were sacrificed on Day 60 or upon reaching maximum tumor burden (volume of 2000 mm^3^).

Immunodeficient female NOD-SCID/IL2γ^−/−^ (NSG) mice (4–6 weeks old) were utilized to establish an animal model of acute myeloid leukemia. The mice were exposed to a radiation dose of 250 cGy to enhance engraftment, following which 2 × 10^6^ THP-1 AML cells containing the luciferase reporter gene were introduced through the tail vein. d-pep-P6 was administered at dosages of 10, 20, and 40 mg/kg, with a pep-NC control group at a dosage of 20 mg/kg every 3 days (*n* = 4 per group). d-Luciferin (Gold Biotechnology Inc., MO) was prepared at a concentration of 15 mg/ml and administered intraperitoneally at a dosage of 10 μl/g of the mouse’s body weight for bioluminescence measurement. Luminescence was measured using an IVIS Spectrum system (PerkinElmer, MA, US), and subsequent image quantification and analysis were performed using Living Image Software (PerkinElmer, MA, US).

### LC–MS/MS

To identify the indicated peptide-interacted proteins, HA-tagged peptides were incubated with THP-1 cells (~10^6^) at 10 μg/ml, 37 °C for 4 h. The cells were then lysed with NP-40 lysis buffer (10 mM Tris-HCl buffered at pH 7.5, 150 mM NaCl, 0.5% NP-40, 1% Triton X-100, 10% Glycerol, 2 mM EDTA, 1 mM NaF, 1 mM Na_3_VO_4_) containing a protease inhibitor cocktail (Sigma-Aldrich, MO, USA). HA-tagged peptides were IP with anti-HA beads, followed by SDS-PAGE to separate peptide-interacted proteins.

The distinct gel bands were cut out and proceeded to in-gel digestion for nanoscale LC–MS/MS to characterize digested peptides as described in a previous report [34]. A data-dependent acquisition mode with a top-ten method was used to operate the mass spectrometer. Full-scan MS spectra were obtained with a target value of 3E6, a resolution of 70,000, and a scan range from 300 to 1800 m/z. Higher collisional dissociation (HCD) tandem MS/MS spectra were obtained with a target value of 1E6, a resolution of 17,500, and a normalized collision energy of 25%. Unknown charges or charges lower than two and higher than eight were excluded. The data were processed using the Agilent MassHunter Workstation Software (version B.01.04) and annotated by PEAKS Studio to identify corresponding proteins.

### siRNA transfection and quantitative real time-PCR

THP-1 cells were seeded into a 96-well plate (1 × 10^4^ per well). After 24 h, the cells were transfected with siRNAs at a final concentration of 200 nM using Lipofectamine RNAiMAX (Invitrogen), following the supplier’s instructions. The siRNA sequences were as follows: siRNA-TLR-2, 5′-CUAGA GUUCU CCUUG GAAA-3′. After 48 h, cell lysis was performed using TRIzol reagent (Invitrogen), and total RNAs were extracted. Subsequently, RNA reverse transcription was performed using the PrimeScript RT reagent kit (TaKaRa), followed by quantitative RT-PCR on a Bio-Rad CFX96 real-time PCR detection system (Bio-Rad) with SYBR Premix Ex Taq (TaKaRa). The primers used for quantitative RT-PCR are listed in Table 4.

**Table 4:** Primer sequences for quantitative RT-PCR.

Target gene	Forward primer (5′–3′)	Reverse primer (5′–3′)
β-actin	GCCAGCAGGTCTCATATCTCG	TTGACGACTAGTGCCGTGGGT
HSP90AA1	AAAAGTTGAAAAGGTGGTTGTGT	GTCAGGGTTTATCTCCAGGTGT
HSP90AB1	GGCCGGGGTACCAAAGTGAT	TCGCCGGGGAATGAAGAG
TLR-2	GATCTCTCAGCGAAGAAACGT	ATTCTGCACGCGTACTCCG
CD33	TGGCCACAAACAAGCTAGATC	GGTTTTTGGAGTGGCCGGGT
CD44	GCACTTCAGGAGGTTACATC	ATCCATGAGTGGTATGGGAC
HMGB1	AATTTCACATAGCCCACTTACATTTACA	TTGATTCTAATAATCCCATGCTTTGA

### Co-immunoprecipitation (Co-IP)

For Co-IP, we synthesize the indicated peptides with an HA tag. The THP-1 cells (~10^6^) were incubated with the peptide at 10 μg/ml, 37 °C for 4 h. The cells were then washed twice with ice-cold PBS and lysed in 500 μL RIPA buffer (50 mM Tris-HCl pH 7.4, 150 mM NaCl, 1% Triton X-100, 1% sodium deoxycholate, 0.1% SDS) containing a protease inhibitor cocktail (Sigma-Aldrich, MO, USA). The lysate was centrifuged at 13,000×*g* for 30 min at 4 °C. To extract the peptide-TLR-2 protein complex, the cell lysate was incubated with 25 μl HA beads (Sigma-Aldrich, MO, USA) or control beads overnight at 4 °C on a rocker. Thereafter, the sample was precipitated by centrifugation at 3000 × *g* for 5 min and washed four times with RIPA buffer to remove nonspecifically bound proteins. The immune complex was tested by Western-blot with TLR-2 antibody (#bs-1019R, Bioss, 1:1000), while anti-GAPDH (#5174, CST, 1:1000) was used as a control.

### Human CD34^+^ short-term culture assay

Peripheral blood mononuclear cells (PBMCs) were extracted from whole blood using Ficoll gradient separation. CD34^+^ T cells were isolated from PBMCs through positive selection utilizing the CD34 Isolation Kit II (Miltenyi). The cells were then cultivated in RPMI 1640 medium supplemented with 100 IU/ml penicillin, 100 μg/ml streptomycin (Sigma), along with 10% FBS (Gibco), and maintained at 37 °C under 5% CO_2_.

### Long-term culture-initiating cell assay

CD34^+^ sorted blasts are cultured on MS5 mouse bone marrow stromal cells using Gartner’s media, which is aMEM (Thermo Fisher) containing 12.5% FCS, 12.5% horse serum (Thermo Fisher), 1% penicillin/streptomycin, 57.2 mmol/L β-mercaptoethanol (Sigma-Aldrich), and 1 mmol/L hydrocortisone (Sigma-Aldrich). This medium is further supplemented with 20 ng/mL thrombopoietin (MedChemExpress), IL3 (Gibco), and G-CSF (Invitrogen). The experiment is performed in duplicate per patient sample.

### Colony-formation assay

Approximately 5000 cells (THP-1, CD34^+^ cells derived from either AML or healthy donors) were cultured with 10 μg/ml d-pep-P6 in 2.6% methylcellulose medium (H4100) supplemented with 10% FBS. Following 15 days of incubation, the number of colonies consisting of more than 50 cells was quantified using an inverted microscope.

### Statistics

Statistical analysis was performed with GraphPad Prism 7. For data with a normal distribution, we used a Student’s *t*-test, and a nonparametric exact Wilcoxon signed-rank test was used to compare data that were not normally distributed. For multiple comparisons (including multiple two-group comparisons shown in the same panel), a one-way or two-way ANOVA (for parametric data) followed by Bonferroni’s correction (only two groups were compared), Dunnett’s test (all groups were compared to one control group), Tukey’s multiple comparison test (all groups were compared to each other), or a Kruskal–Wallis test (for nonparametric data) followed by Dunn’s multiple comparison test were used. Correlation was estimated by Pearson correlation coefficients (for parametric data). *P* values < 0.05 were considered statistically significant.

### Supplementary information

Supplemental Figure

## Data Availability

All data generated or analyzed during this study are included in this published article and its supplementary information files.

## References

[1] Döhner H, Weisdorf DJ, Bloomfield CD (2015). Acute myeloid leukemia. N Engl J Med.

[2] Daver N, Wei AH, Pollyea DA, Fathi AT, Vyas P, DiNardo CD (2020). New directions for emerging therapies in acute myeloid leukemia: the next chapter. Blood Cancer J.

[3] Tallman MS, Wang ES, Altman JK, Appelbaum FR, Bhatt VR, Bixby D (2019). Acute myeloid leukemia, version 3.2019, NCCN clinical practice guidelines in oncology. J Natl Compr Cancer Netw.

[4] Kucukyurt S, Eskazan AE (2019). New drugs approved for acute myeloid leukaemia in 2018. Br J Clin Pharm.

[5] Warrell RP, Frankel SR, Miller WH, Scheinberg DA, Itri LM, Hittelman WN (1991). Differentiation therapy of acute promyelocytic leukemia with tretinoin (all-trans-retinoic acid). N Engl J Med.

[6] Kantarjian H, Sawyers C, Hochhaus A, Guilhot F, Schiffer C, Gambacorti-Passerini C (2002). Hematologic and cytogenetic responses to imatinib mesylate in chronic myelogenous leukemia. N Engl J Med.

[7] Estey EH (2018). Acute myeloid leukemia: 2019 update on risk-stratification and management. Am J Hematol.

[8] Omidfar K, Daneshpour M (2015). Advances in phage display technology for drug discovery. Expert Opin Drug Dis.

[9] Smith GP (1985). Filamentous fusion phage: novel expression vectors that display cloned antigens on the virion surface. Science.

[10] Alfaleh MA, Alsaab HO, Mahmoud AB, Alkayyal AA, Jones ML, Mahler SM (2020). Phage display derived monoclonal antibodies: from bench to bedside. Front Immunol.

[11] Bosshart H, Heinzelmann M (2016). THP-1 cells as a model for human monocytes. Ann Transl Med.

[12] Feng Z, Xu B (2016). Inspiration from the mirror: D-amino acid containing peptides in biomedical approaches. Biomol Concepts.

[13] Tsao T, Shi Y, Kornblau S, Lu H, Konoplev S, Antony A (2012). Concomitant inhibition of DNA methyltransferase and BCL-2 protein function synergistically induce mitochondrial apoptosis in acute myelogenous leukemia cells. Ann Hematol.

[14] DiNardo CD, Stein EM, de Botton S, Roboz GJ, Altman JK, Mims AS (2018). Durable remissions with ivosidenib in IDH1-mutated relapsed or refractory AML. N. Engl J Med.

[15] Stein EM, DiNardo CD, Fathi AT, Pollyea DA, Stone RM, Altman JK (2019). Molecular remission and response patterns in patients with mutant-IDH2 acute myeloid leukemia treated with enasidenib. Blood.

[16] Schenk T, Chen WC, Göllner S, Howell L, Jin L, Hebestreit K (2012). Inhibition of the LSD1 (KDM1A) demethylase reactivates the all-trans-retinoic acid differentiation pathway in acute myeloid leukemia. Nat Med.

[17] Nachliely M, Sharony E, Kutner A, Danilenko M (2016). Novel analogs of 1, 25-dihydroxyvitamin D2 combined with a plant polyphenol as highly efficient inducers of differentiation in human acute myeloid leukemia cells. J Steroid Biochem.

[18] Madan V, Koeffler HP (2021). Differentiation therapy of myeloid leukemia: four decades of development. Haematol J.

[19] DiNardo C, Lachowiez C (2019). Acute Myeloid Leukemia: from mutation profiling to treatment decisions. Curr Hematol Malig R.

[20] Zeidan AM, Wang R, Wang X, Shallis RM, Podoltsev NA, Bewersdorf JP (2019). Clinical outcomes of older patients (pts) with acute myeloid leukemia (AML) receiving hypomethylating agents (HMAs): a large population-based study in the United States. Blood.

[21] Schlenk RF, Fröhling S, Hartmann F, Fischer JT, Glasmacher A, Del Valle F (2004). Phase III study of all-trans retinoic acid in previously untreated patients 61 years or older with acute myeloid leukemia. Leukemia.

[22] Schlenk RF, Döhner K, Kneba M, Götze K, Hartmann F, Del Valle F (2009). Gene mutations and response to treatment with all-trans retinoic acid in elderly patients with acute myeloid leukemia. Results from the AMLSG Trial AML HD98B. Haematologica.

[23] Ladner RC, Sato AK, Gorzelany J, de Souza M (2004). Phage display-derived peptides as therapeutic alternatives to antibodies. Drug Discov Today.

[24] Ni S, Zhuo Z, Pan Y, Yu Y, Li F, Liu J (2021). Recent progress in aptamer discoveries and modifications for therapeutic applications. ACS Appl Mater Inter.

[25] Arranz-Gibert P, Ciudad S, Seco J, Garcia J, Giralt E, Teixido M (2018). Immunosilencing peptides by stereochemical inversion and sequence reversal: retro-D-peptides. Sci Rep..

[26] Liu M, Li C, Pazgier M, Li C, Mao Y, Lv Y (2010). D-peptide inhibitors of the p53-MDM2 interaction for targeted molecular therapy of malignant neoplasms. Proc Natl Acad Sci USA.

[27] Ramzi M, Khalafi-Nezhad A, Saadi MI, Jowkar Z (2018). Association between TLR2 and TLR4 expression and response to induction therapy in acute myeloid leukemia patients. Int J Hematol Oncol Stem Cell Res.

[28] Ignatz-Hoover JJ, Wang H, Moreton SA, Chakrabarti A, Agarwal MK, Sun K (2015). The role of TLR8 signaling in acute myeloid leukemia differentiation. Leukemia.

[29] Rybka J, Butrym A, Wróbel T, Jazwiec B, Bogucka-Fedorczuk A, Poreba R (2016). The expression of toll-like receptors in patients with B-cell chronic lymphocytic leukemia. Arch Immunol Ther Ex.

[30] Collins S, Ruscetti F, Gallagher R, Gallo R (1979). Normal functional characteristics of cultured human promyelocytic leukemia cells (HL-60) after induction of differentiation by dimethylsulfoxide. J Exp Med.

[31] Okamoto M, Hirai H, Taniguchi K, Shimura K, Inaba T, Shimazaki C (2009). Toll-like Receptors (TLRs) are expressed by myeloid leukaemia cell lines, but fail to trigger differentiation in response to the respective TLR ligands. Br J Haematol.

[32] Zhou L, Shen LH, Hu LH, Ge H, Pu J, Chai DJ (2010). Retinoid X receptor agonists inhibit phorbol-12-myristate-13-acetate (PMA)-induced differentiation of monocytic THP-1 cells into macrophages. Mol Cell Biochem.

[33] Pession A, Martino V, Tonelli R, Beltramini C, Locatelli F, Biserni G (2003). MLL-AF9 oncogene expression affects cell growth but not terminal differentiation and is downregulated during monocyte-macrophage maturation in AML-M5 THP-1 cells. Oncogene.

[34] Ma X, Yang T, Luo Y, Wu L, Jiang Y, Song Z (2019). TRIM28 promotes HIV-1 latency by SUMOylating CDK9 and inhibiting P-TEFb. Elife.

