# Supplementary material for: Generation of a new therapeutic d-peptide that induces the differentiation of acute myeloid leukemia cells through A TLR-2 signaling pathway
Source: Cell Death Discov. 2024 Jan 26;10:51. doi: 10.1038/s41420-024-01822-w (PMC10810823; doi:10.1038/s41420-024-01822-w)
Supplement: Supplementary file 1 — Supplemental Figure [file 41420_2024_1822_MOESM1_ESM.pdf]

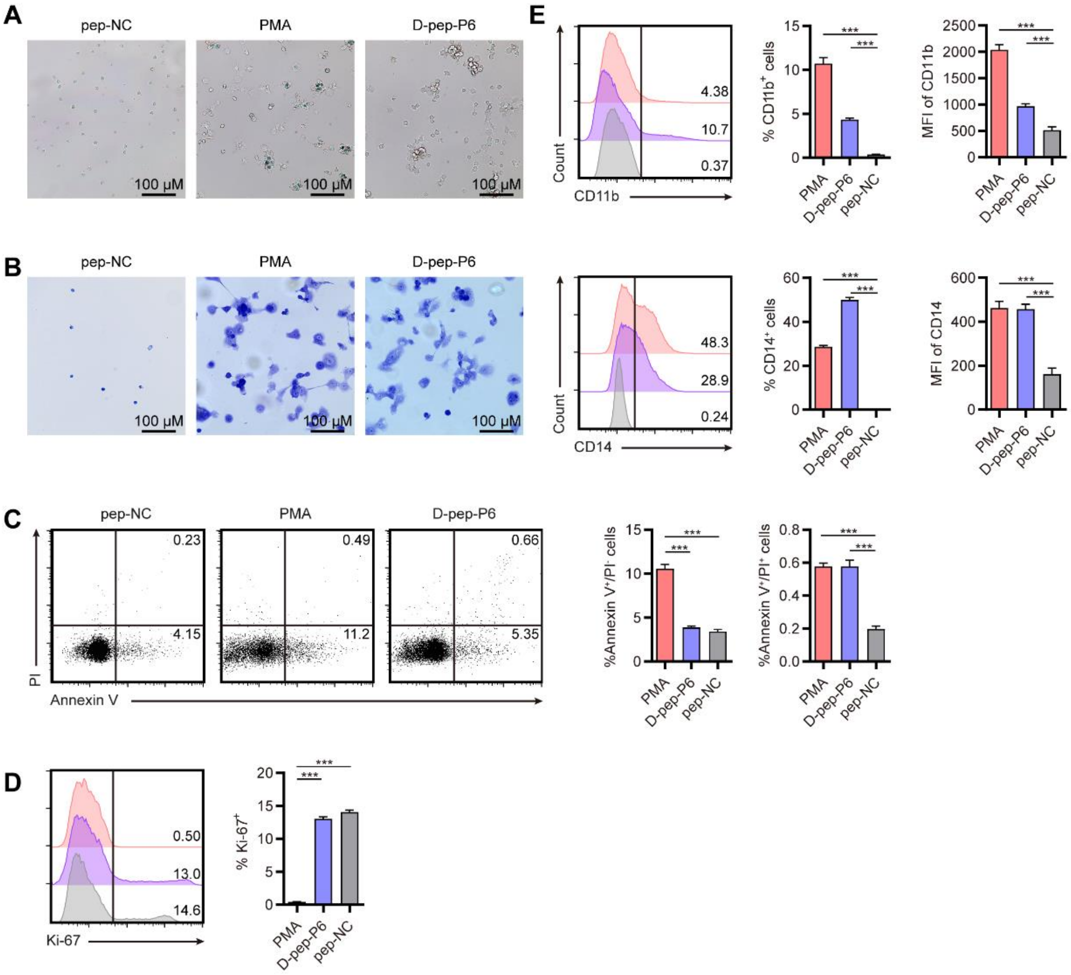

Supplemental Figure 1. D-pep-P6 could induce differentiation of HL-60 cells. HL-60 cells were stimulated with D-pep-P6 (10 μg/ml), pep-NC (10 μg/ml), or PMA for 48 hours. (A) The suspended cells were gently removed through a PBS wash. The morphological features were then visualized by phase contrast microscopy. (B) Phenotype was detected by May-Grünwald Giemsa staining. (C and D) Flow cytometry was utilized to assess apoptosis via Annexin V/propidium iodide (PI) staining (C) and proliferation via Ki-67 staining (D). (E) Cell surface marker CD11b and CD14 were detected by FACS. Each bar represents mean and SEM from three independent experiments in triplicate. ****P*< 0.001 (Kruskal-Wallis test). MFI, median fluorescent intensity. Data are from 3 independent experiments.

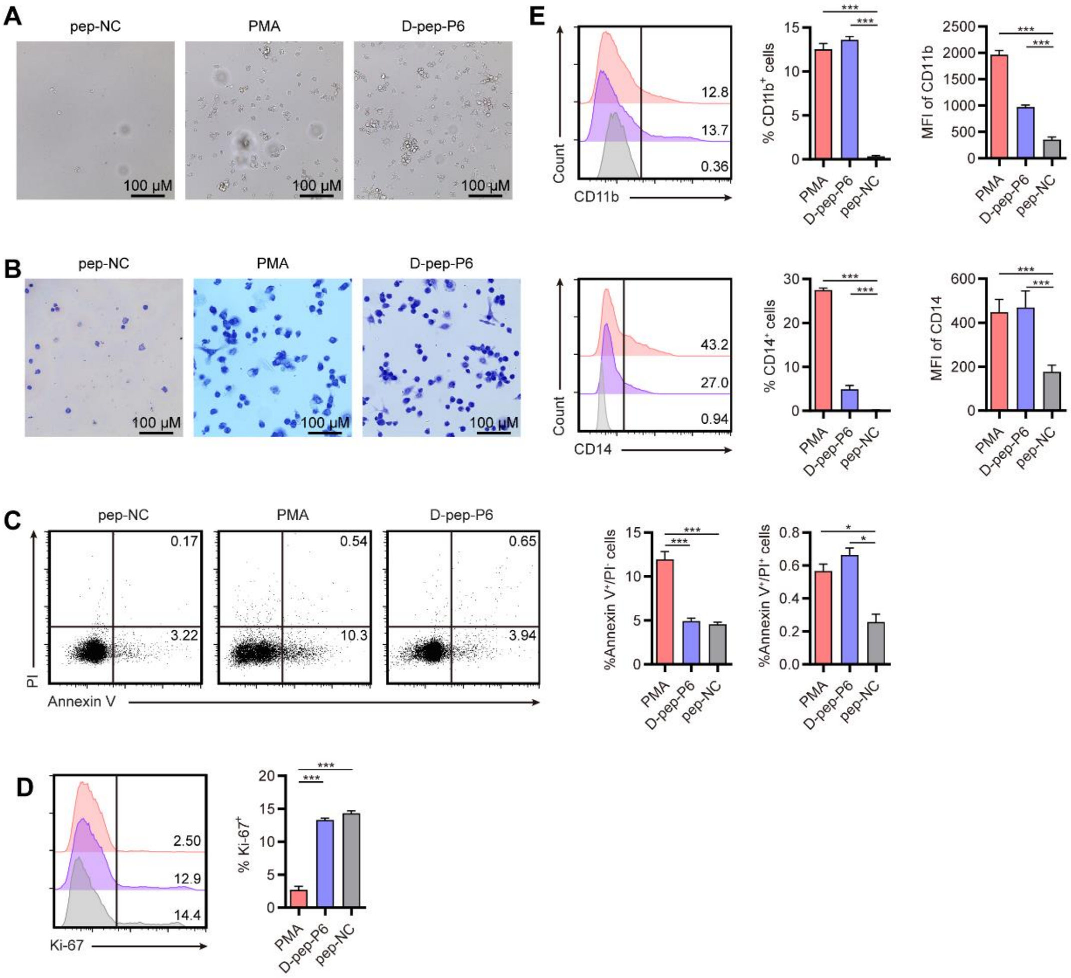

Supplemental Figure 2. D-pep-P6 could induce differentiation of U937 cells. U937 cells were stimulated with D-pep-P6 (10 μg/ml), pep-NC (10 μg/ml), or PMA for 48 hours. (A) The suspended cells were gently removed through a PBS wash. The morphological features were then visualized by phase contrast microscopy. (B) Phenotype was detected by May-Grünwald Giemsa staining. (C and D) Flow cytometry was utilized to assess apoptosis via Annexin V/propidium iodide (PI) staining (C) and proliferation via Ki-67 staining (D). (E) Cell surface marker CD11b and CD14 were detected by FACS. Each bar represents mean and SEM from three independent experiments in triplicate. *P< 0.05, ***P< 0.001 (Kruskal-Wallis test). MFI, median fluorescent intensity. Data are from 3 independent experiments.

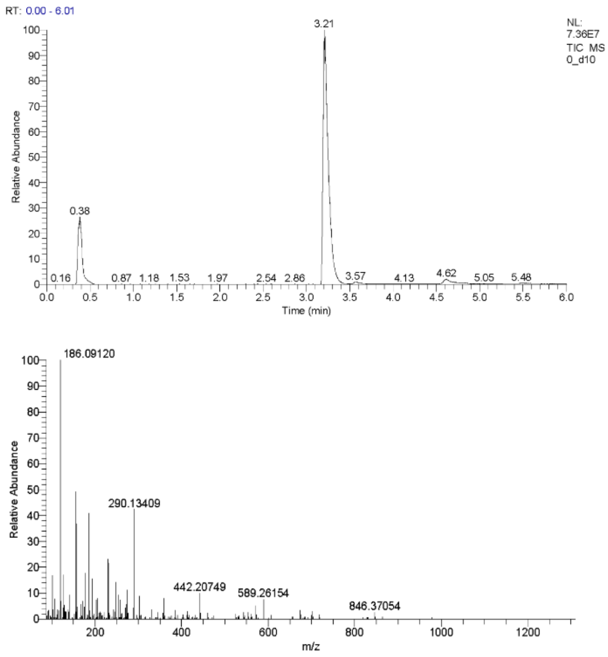

Supplemental Figure 3. Characterizations of D-pep-P6 by HPLC chromatograms (λ=214 nm) and ESI-MS. HPLC conditions: 0.1% formic acid aqueous solution over 10 min on a XB-C18 (50 × 2.1 mm) column.

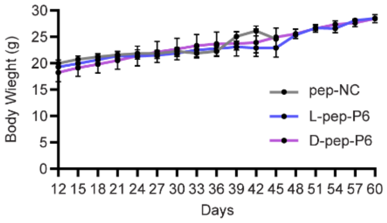

Supplemental Figure 4. Body weight changes of THP-1 tumor-bearing mice were monitored over the course of 60 days. Statistical analyses were performed to compare the body weight between the 20 mg/kg L-/D-pep-P6 and 20 mg/kg pep-NC treated groups in NOD/SCID THP-1 tumor-bearing mice (n = 16 mice per group).

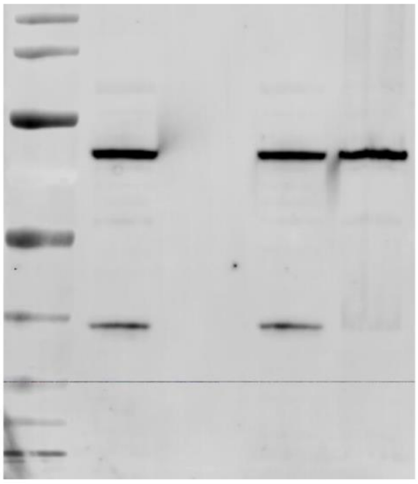

Supplemental Figure 5. Full length Western blot analysis of Figure 5B. Co-IP D-pep-P6 with TLR-2 used anti-TLR-2 mAb.
